# MFM and PEEM observation of micrometre-sized magnetic dot arrays fabricated by ion-microbeam irradiation in FeRh thin films

**DOI:** 10.1107/S0909049511054057

**Published:** 2012-01-31

**Authors:** K. Aikoh, A. Tohki, T. Matsui, A. Iwase, T. Satoh, K. Takano, M. Kohka, Y. Saitoh, T. Kamiya, T. Ohkochi, M. Kotsugi, T. Nakamura, T. Kinoshita

**Affiliations:** a Department of Materials Science, Osaka Prefecture University, 1-1 Gakuen-cho, Naka-ku, Sakai, Osaka 599-8531, Japan; b Research Organization for the 21st Cemtury, Osaka Pefecture University, 1-1 Gakuen-cho, Sakai, Osaka 599-8531, Japan; c Takasaki Advanced Radiation Research Institute, Japan Atomic Energy Agency, Takasaki, Gumma 370-1292, Japan; d Japan Synchrotron Radiation Research Institute, Sayo, Hyogo 679-5198, Japan; e CREST-JST, Kawaguchi, Saitama 332-0012, Japan

**Keywords:** energetic ion-beam irradiation, first-order magnetic phase transition, magnetic patterning, MFM, XMCD–PEEM

## Abstract

FeRh thin films were irradiated with a 10 MeV iodine ion microbeam to produce micrometre-scale ferromagnetic microstructures by modifying the local magnetic character from antiferromagnetism to ferromagnetism using ion-microbeam irradiation. Two-dimensional magnetic dot arrays of dimensions ∼2 µm × 4 µm as well as 10 µm × 10 µm were successfully produced on the FeRh surface, which was confirmed by magnetic force microscopy (MFM). The results of photoemission electron microscopy (PEEM) combined with X-ray magnetic circular dichroism reveal that the easy axis of the magnetization of the ion-beam-irradiated ferromagnetism in the FeRh thin films lies in the film planes along the 〈001〉 direction of the MgO substrates.

## Abstract 2

Micrometre-size lateral magnetic modulations were fabricated in FeRh thin films by ion-microbeam irradiation. Their magnetic domain structures were characterized by XMCD–PEEM.

## 1. Introduction

Intermetallic compounds of FeRh with B2 (CsCl-type) structure have attracted interest owing to their fundamental magnetic properties such as first-order phase transitions from anti-ferromagnetic (AF) to ferromagnetic (FM) near room temperature and to their potential technological applications (Fallot & Hocart, 1939 ▶; Kouvel, 1966 ▶; Thiele *et al.*, 2003 ▶; Hashi *et al.*, 2004 ▶). In our previous studies we reported that the deposited energy through elastic collisions by high-energy ion-beam irradiation induced the FM state in FeRh bulk and film samples below room temperatures where they were originally in the AF state (Zushi *et al.*, 2007 ▶; Kosugi *et al.*, 2009 ▶; Fujita *et al.*, 2010*a* ▶). In addition, we have successfully produced micrometre-size lateral magnetic modulations by using the technique of ion-microbeam irradiation (Fujita *et al.*, 2010*b* ▶). Here, the ion microbeam is a technique for focusing MeV energy ions down to a few micrometres or less in lateral size at a target sample, and for introducing localized high-density energy deposition with a high-aspect ratio (Ishii *et al.*, 2001 ▶).

In recent years patterned magnetic thin films have been of interest, not only because of the potential for technological applications, such as magnetic recording and magnetoresistive devices, but also because of the unique magnetic behaviour as the lateral dimensions are reduced (Terris & Thomson, 2005 ▶; Zhu *et al.*, 2000 ▶; Jiang *et al.*, 2004 ▶). If such laterally modulated magnetic structures with AF and FM micrometre-sized areas can readily be fabricated in FeRh alloys, new magnetic phenomena as well as potential applications of FeRh to new devices can be expected.

During the course of the present studies we have attempted to fabricate lateral magnetic modifications of various sizes, shapes and magnetic natures on FeRh thin film by high-energy ion-microbeam irradiation. Two-dimensional distributions of the deposited energy density at the sample surface can be determined by controlling the scanning mode and current of the high-energy ion microbeam. With this technique we attempted to make micrometre-sized modulations of the in-plane magnetic state of FeRh films. In this paper we report the characterization of the magnetic modulations by using a magnetic force microscopy (MFM) as well as a photoemission electron microscopy (PEEM) technique at a synchrotron X-ray facility.

## 2. Experimental procedure

Fe_47_Rh_53_ thin films of thickness ∼80 nm were deposited on MgO (100) substrates at 973 K by means of ion-beam sputtering from an alloy target of Fe_50_Rh_50_. After the deposition, the films were annealed at 1073 K for 4 h under a pressure of 4 × 10^−4^ Pa to obtain an ordered FeRh (B2) phase. In order to determine the appropriate amount of ion fluence to produce the micrometre-sized magnetic modification of the FeRh thin film samples, the FeRh thin film was irradiated uniformly with 10 MeV iodine ions by using a tandem accelerator at Takasaki Ion Accelerators for Advanced Radiation Application (TIARA) of the Japan Atomic Energy Agency (JAEA). The samples were irradiated at room temperature with the ion fluence ranging between 2 × 10^12^ and 1 × 10^14^ cm^−2^. Before and after irradiation, the magnetic properties of the samples were measured using a superconducting quantum interference device magnetometer. The magnetic hysteresis (M-H) curves were measured in the range −6 to 6 kOe at 20 K. The crystal structure of the samples was also characterized using the X-ray diffraction (XRD) method and extended X-ray absorption fine structure (EXAFS) spectroscopy.

To realise the lateral magnetic modifications on the sample surface, ion-microbeam irradiation was performed by using a 3 MV tandem accelerator at TIARA-JAEA. The focused 10 MeV iodine ion microbeam, which has a rectangular shape of 2 µm × 4 µm, was scanned on the FeRh thin-film surface. By using such a micro-ion probe, several different magnetic patterns can be drawn: one typical example is 10 µm × 10 µm squares with 20 µm intervals in the *x* and *y* directions over the 200 µm × 200 µm area. The *x* and *y* directions were arranged along the 〈001〉 directions of the MgO substrates.

After irradiations, the surface roughness and the magnetic state were observed by means of magnetic force microscopy (MFM; Shimadzu SPM9600) with the applied magnetic field perpendicular to the film surface by using a fairly strong Nd-Fe-B permanent magnet. Hence, the obtained magnetic images do not contain information on the in-plane magnetic domain structure. In contrast, photoemission electron microscopy combined with X-ray magnetic circular dichroism (XMCD–PEEM) is a potential technique for studying more details of the magnetic domain structures at sample surfaces (Kinoshita, 2002 ▶). Since XMCD–PEEM provides information on the magnetization vector projected onto the incident direction of the synchrotron radiation beam, it is possible to obtain all three independent components of the magnetization vector by rotating the sample. The magnetic imaging experiment was carried out at the soft X-ray undulator beamline BL25SU at SPring-8, where a conventional PEEM system was installed. The incident angle of the synchrotron radiation to the sample surface is 30°. The XMCD–PEEM images were recorded at the photon energy of the Fe *L*_3_ absorption edge (707 eV) at room temperature. Magnetic images were obtained by taking the difference between two images taken with right- and left-circularly polarized light. Bias magnetic field from a Nd-Fe-B permanent magnet was applied along the [001] direction for the MgO substrates of all the samples for a short time before the PEEM measurements.

## 3. Results and discussion

**Figure 1:**
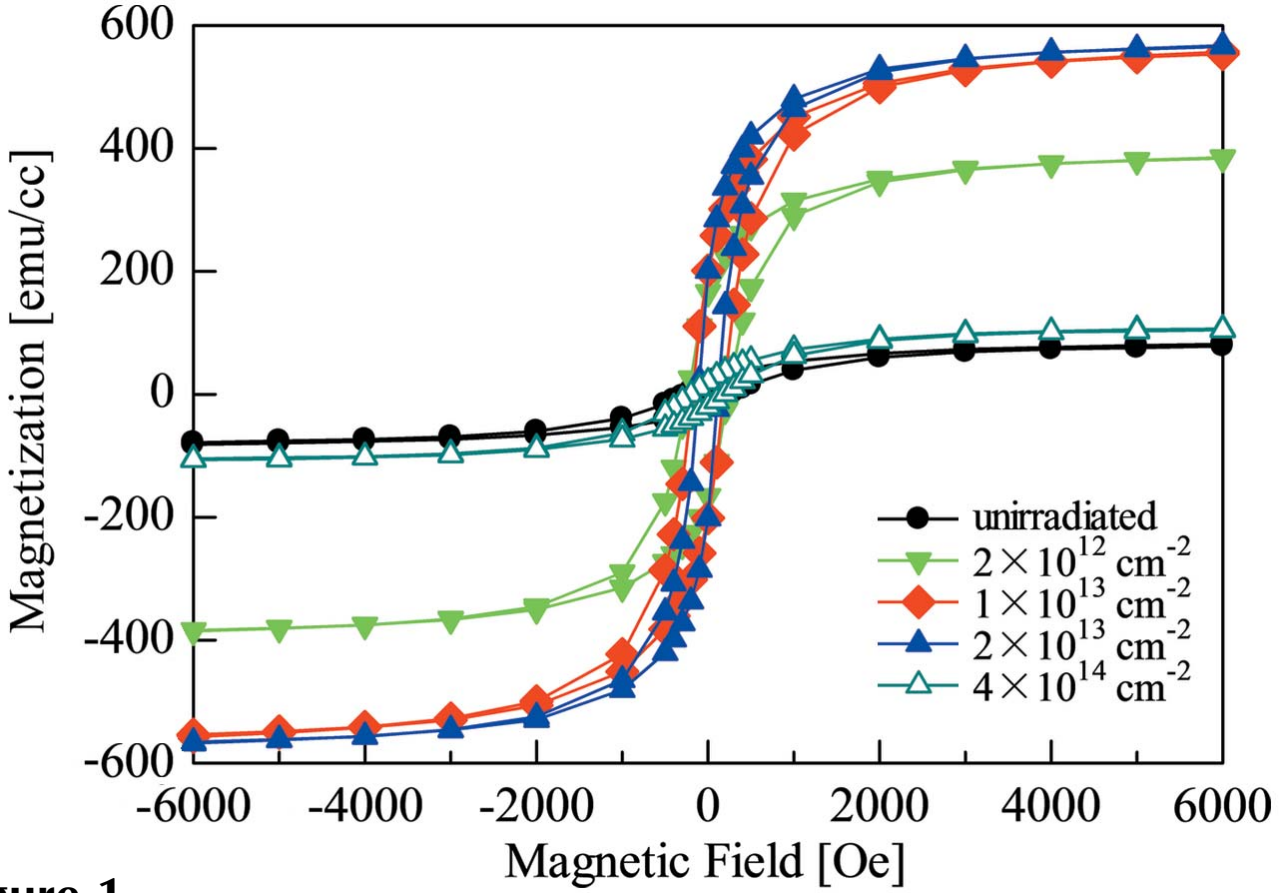
M-H curves measured at 20 K for the unirradiated sample and for the samples irradiated with a 10 MeV iodine ion beam.

Magnetic hysteresis loops measured at 20 K in the unirradiated and irradiated samples with various ion fluences of 2 × 10^12^ cm^−2^ to 1 × 10^14^ cm^−2^ are shown in Fig. 1 ▶. Even in the unirradiated sample a small magnetization of about 70 emu cc^−1^ was observed: this is caused by lattice defects in the B2 phase that exist prior to the ion exposure (Fujita *et al.*, 2010*a* ▶). As can be clearly seen in the figure, the ion irradiation increases the magnetization of the films. The maximum value of saturation magnetization, 570 emu cc^−1^, is obtained in the sample irradiated with an ion fluence of 1–2 × 10^13^ cm^−2^. Further increases in the ion fluence decrease the magnetization of the films. The structural characterizations by XRD and EXAFS show that the films maintain the B2 ordered structure up to ion fluences of 1–2 × 10^13^ cm^−2^ but decompose by further influence to non-magnetic random face-centred cubic (A1) phases, as shown in the figure (Kosugi *et al.*, 2011 ▶). Hence, the ion fluence required to produce the largest magnetization was successfully determined for the series of the present samples, which directly corresponded to the suitable irradiation time for ion-microbeam irradiation for the magnetic patterning.

**Figure 2:**
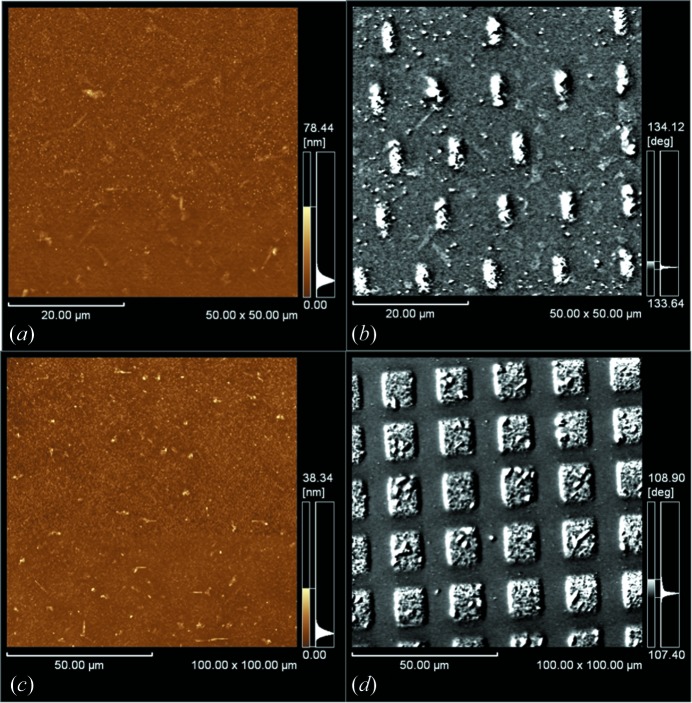
Micrometre-sized patterning in the FeRh thin film using a 10 MeV iodine ion microbeam: (*a*) and (*c*) topographic (AFM) images; (*b*) and (*d*) MFM images.

Thereafter we performed ion-microbeam irradiation. The topographic (atomic force microscopic, AFM) and MFM images of a typical microbeam-irradiated region are shown in Figs. 2(*a*) and 2(*b*) ▶, respectively. The AFM image in Fig. 2(*a*) ▶ shows that the ion microbeam does not create any topographic modification at the FeRh surface, while a rectangular array is seen with approximate 2.1 µm × 4.4 µm bright rectangles at designated regular intervals in Fig. 2(*b*) ▶. Since the degree of brightness of a MFM image corresponds to the strength of force operating between the MFM cantilever and the sample surface, a ferromagnetic area can be seen much brighter than that of anti-ferromagnetic or non-magnetic areas in the MFM images. Accordingly, the observed MFM image clearly shows the presence of the micrometre-sized magnetic modification. Since each rectangle was made by fixed ion-beam irradiation, the size corresponds to that of an ion microbeam.

Another ferromagnetic pattern that was produced by the 10 MeV iodine microbeam with a different shape and different interval is shown in Figs. 2(*c*) and 2(*d*) ▶. The AFM image in Fig. 2(*c*) ▶ does not show any structure as in Fig. 2(*a*) ▶. As shown in Fig. 2(*d*) ▶, the magnetic pattern differs from that in Fig. 2(*a*) ▶; each ferromagnetic area is a bright square of about 11.8 µm × 12.8 µm in size. These results imply that the energetic ion microbeam can be used as a tool to produce micrometre-sized modulations of lateral magnetic states of FeRh films.

**Figure 3:**
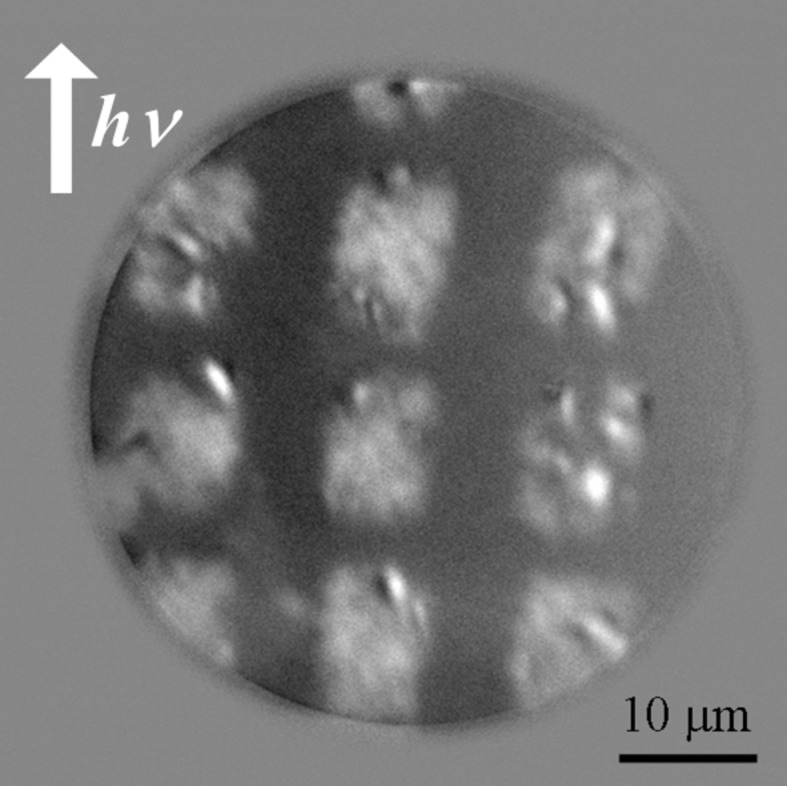
Magnetic domain image of the FeRh thin film irradiated with a 10 MeV ion microbeam at room temperature.

**Figure 4:**
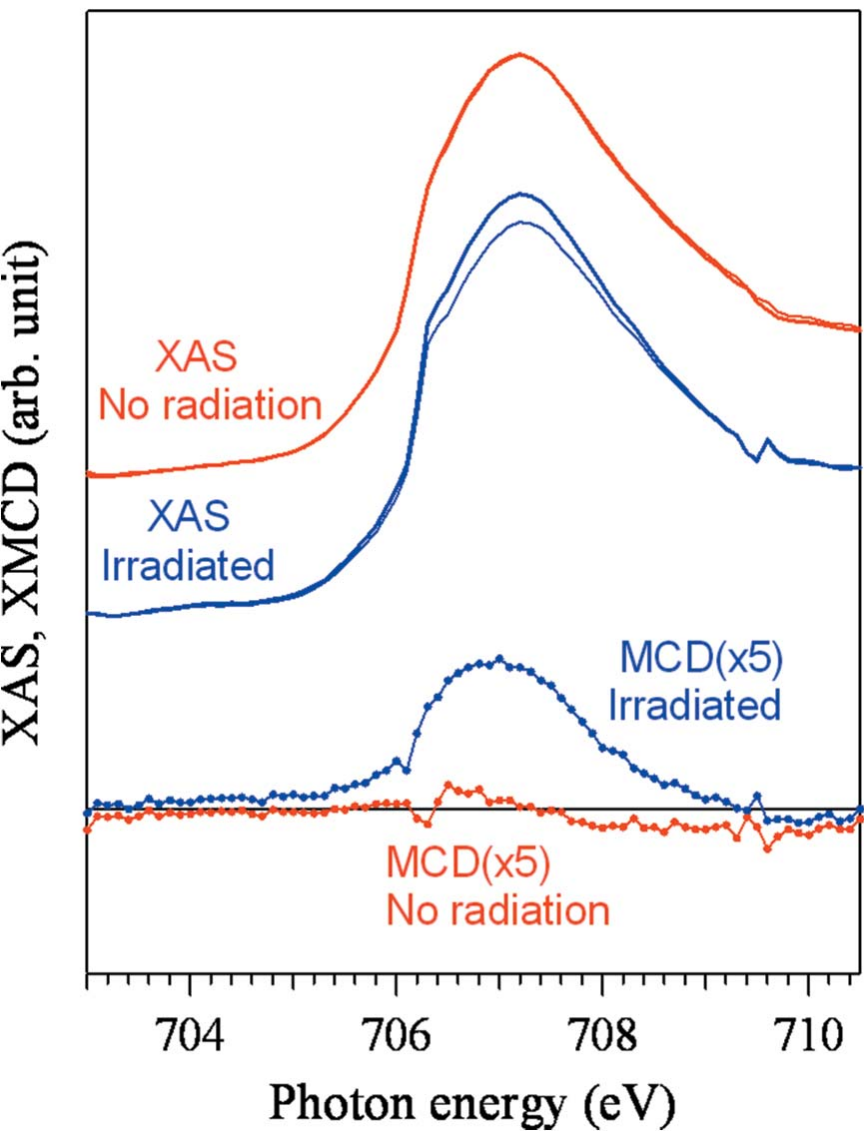
XAS and XMCD spectra measured from the irradiated and unirradiated regions of the samples.

Fig. 3 ▶ shows the XMCD–PEEM images of the FeRh thin film with a field of view of 50 µm with lateral magnetic modulations. The incident direction of the synchrotron radiation beam was parallel to the [001] direction of the MgO substrate. The bright squares are the same as those observed in Fig. 2(*d*) ▶, indicating that the observed bright regions correspond to the irradiated regions with the ion microbeam. Fig. 4 ▶ shows the local XAS and XMCD spectra at the photon energy of the Fe *L*_3_ absorption edge (707 eV) taken from the corresponding squarely irradiated region, as well as from the unirradiated region. The clear XMCD signal can be observed only for the spectrum taken from the irradiated regions. Accordingly, the square regions with the same dimensions as the irradiated region have a definite magnetic moment, which simply means they are FM.

**Figure 5:**
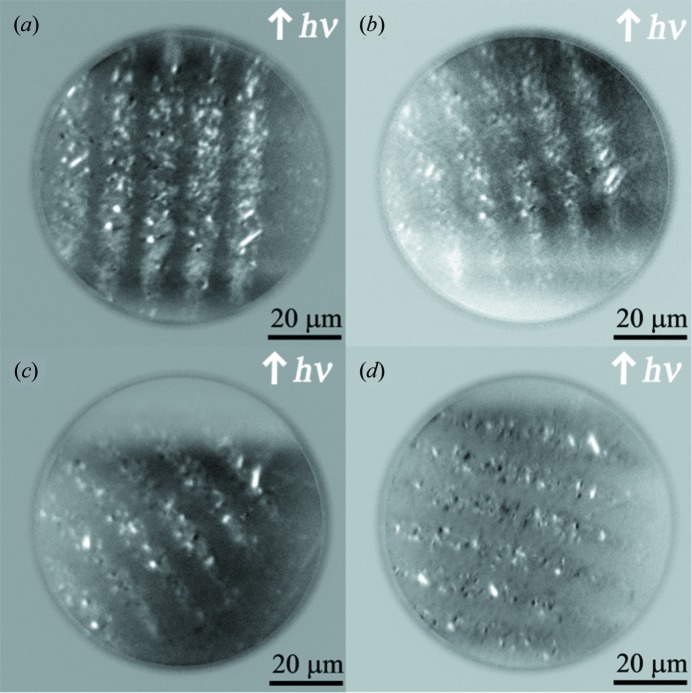
Magnetic domain image of the FeRh thin film irradiated with a 10 MeV ion microbeam taken at room temperature at azimuthal angles of (*a*) 0°, (*b*) 22.5°, (*c*) 45° and (*d*) 80° between the incident synchrotron radiation beam direction and the [001] direction of the MgO substrate.

To confirm the direction of the magnetization seen in Fig. 3 ▶, we show magnetic domain images of the FeRh film sample in Figs. 5(*a*)–5(*d*) ▶ at azimuthal angles of 0°, 22.5°, 45° and 80°, respectively, to the incident synchrotron radiation beam direction, *i.e.* the [001] direction of the MgO substrate. The stripe-like magnetic patterns can be observed in Fig. 5(*a*) ▶, which is slightly different from the square dot pattern in Fig. 3 ▶. This may be due to the insufficient alignment of PEEM optics in this observation. Still, the domain images show that the brightest contrast is obtained at an azimuthal angle of 0°, and that the contrast becomes darker with increasing azimuthal angle up to 80°. Since the contrast in the XMCD–PEEM images reflects the magnetization vector projected along the incident synchrotron radiation beam direction, these experimental results suggest that most of the residual magnetization directs parallel to the [001] direction of the MgO substrates. This is because the sample was exposed to the magnetic field along the [001] direction of MgO before installation in the PEEM chamber. Hence, we conclude that the easy axis of the magnetization of the samples lies in the film planes along the 〈001〉 direction of the MgO substrates, neither along the 〈011〉 nor 〈111〉 directions, although several minor regions (the small bright areas remain unchanged with the sample rotation) may have a perpendicular magnetic component. These results suggest that the FeRh film almost epitaxically grows on the MgO substrates. Further quantitative analyses for the XMCD–PEEM images as well as a detailed structural characterization of the FeRh films are now in progress.

**Figure 6:**
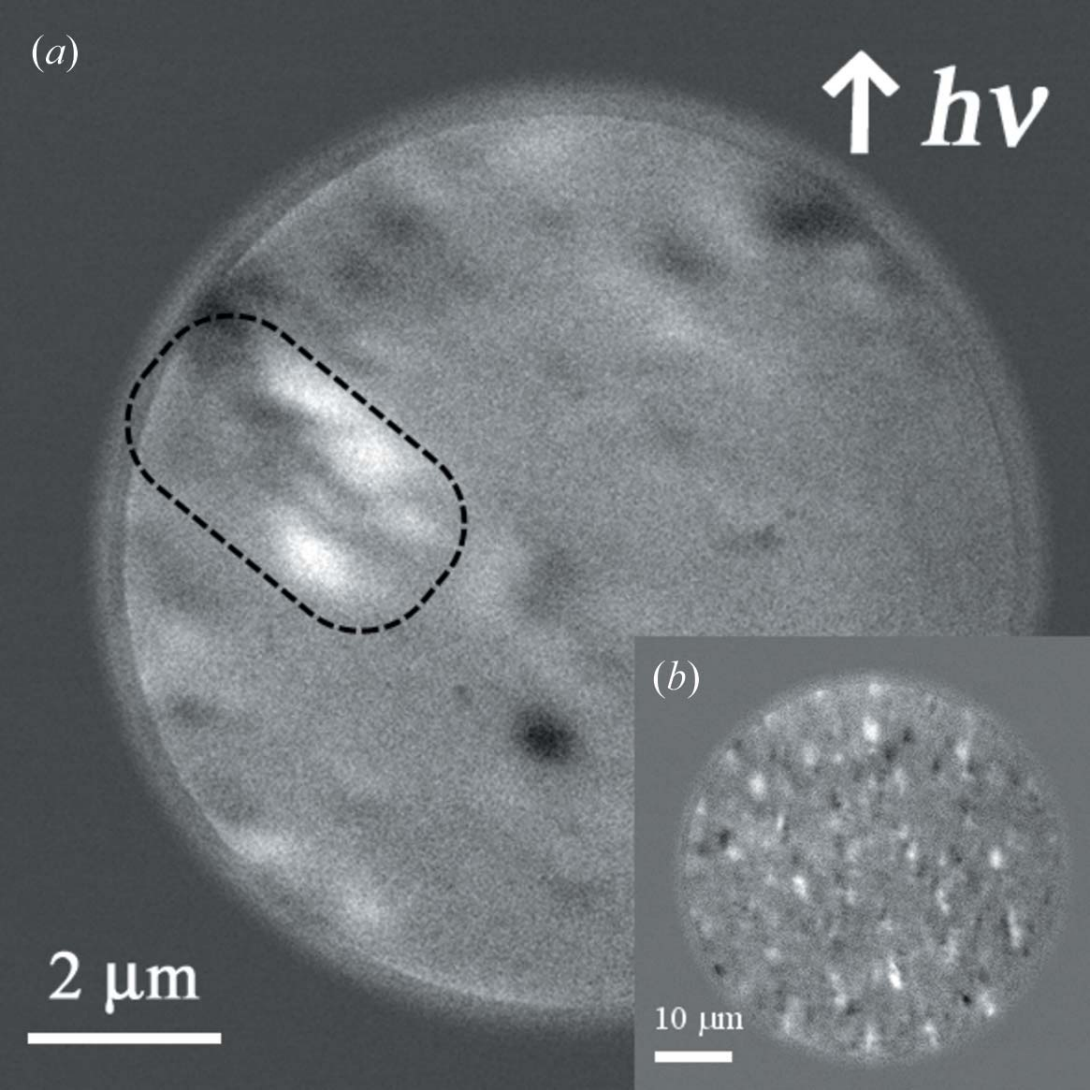
Magnetic domain image of 10 µm field of view for the FeRh thin film irradiated with a 10 MeV ion microbeam at room temperature (*a*). (*b*) Lower magnified image.

In addition, we could also observe the smaller magnetic patterned image with a 10 µm field of view, as shown in Fig. 6(*a*) ▶. A lower magnified image is also inset in Fig. 6(*b*) ▶. The observed dot pattern is almost equivalent to the same dimension as the ion microbeam profile (2.1 µm × 4.4 µm) as shown in the MFM image in Fig. 2(*b*) ▶. The ellipsoidal region encircled by the dashed line with white and black contrast in Fig. 6(*a*) ▶ can be attributed to the magnetic domain, although the image is not well resolved, possibly due to the imperfect tilt correction, inhomogeneous surface electric field, the unsuitable sample shape or size for the PEEM observation, *etc.* We expect that the local magnetic domain structure can be quantitatively discussed by slightly improving the experimental conditions.

## 4. Conclusions

We have successfully produced the micrometre-sized lateral magnetic modulation structure in the FeRh thin film by energetic ion-microbeam irradiation. The MFM image of the particular microbeam irradiated region clearly indicated bright dot arrays with the same dimensions as the microbeam. On the other hand, no topological contrast could be seen in the AFM images in the same region. The analysis of the contrast in XMCD–PEEM images reflecting the magnetization vector projected along the incident synchrotron radiation beam direction indicated that the easy axis of the magnetization of the samples lies in the film planes along the 〈001〉 direction of the MgO substrates.
